# Natural killer cytotoxicity in myalgic encephalomyelitis/chronic fatigue syndrome (ME/CFS): a multi-site clinical assessment of ME/CFS (MCAM) sub-study

**DOI:** 10.1186/s12967-023-03958-2

**Published:** 2023-04-03

**Authors:** Troy D. Querec, Jin-Mann S. Lin, Yang Chen, Britany Helton, Andreas M. Kogelnik, Nancy G. Klimas, Daniel L. Peterson, Lucinda Bateman, Charles Lapp, Richard N. Podell, Benjamin H. Natelson, Elizabeth R. Unger, the MCAM Study Group, Elizabeth Unger, Jin-Mann Sally Lin, Monica Cornelius, Irina Dimulescu, Elizabeth Fall, Maung Khin, Mangalathu Rajeevan, Jennifer Bland, Patricia Jeys, Veronica Parkinson, Wendy Springs, Nancy Klimas, Elizabeth Balbin, Jeffry Cournoyer, Melissa Fernandez, Shuntae Parnell, Precious Leaks-Gutierrez, Benjamin Natelson, Michelle Blate, Gudrun Lange, Sarah Khan, Diana Vu, Andreas Kogelnik, Joan Danver, David Kaufman, Macy Pa, Catt Phan, Sophia Taleghani, Richard N Podell, Trisha Fitzpatrick, Beverly Licata, Daniel Peterson, Elena Lascu, Gunnar Gottschalk, Marco Maynard, Janet Smith

**Affiliations:** 1 Division of High-Consequence Pathogens and Pathology, Centers for Disease Control and Prevention, Atlanta, GA USA; 2 Open Medicine Clinic, Mountain View, CA USA; 3 Institute for Neuro Immune Medicine, Miami, FL USA; 4 Sierra Internal Medicine, Incline Village, NV USA; 5 The Bateman Horne Center, Salt Lake City, UT USA; 6 Hunter-Hopkins Center, Charlotte, NC USA; 7 Richard N. Podell Medical, Summit, NJ USA; 8 Department of Neurology, Mount Sinai Beth Israel, New York, NY USA

**Keywords:** Natural killer cell (NK), Cytotoxicity, Comorbidity, Multimorbidity (multiple medical conditions), Multicenter study, Myalgic encephalomyelitis/chronic fatigue syndrome (ME/CFS)

## Abstract

**Background:**

Myalgic encephalomyelitis/chronic fatigue syndrome (ME/CFS) is a multisystem illness characterized by substantial reduction in function accompanied by profound unexplained fatigue not significantly relieved by rest, post-exertional malaise, and other symptoms. Reduced natural killer (NK) cell count and cytotoxicity has been investigated as a biomarker for ME/CFS, but few clinical laboratories offer the test and multi-site verification studies have not been conducted.

**Methods:**

We determined NK cell counts and cytotoxicity in 174 (65%) ME/CFS, 86 (32%) healthy control (HC) and 10 (3.7%) participants with other fatigue associated conditions (ill control [IC]) from the Multi-Site Clinical Assessment of ME/CFS (MCAM) study using an assay validated for samples shipped overnight instead of testing on day of venipuncture.

**Results:**

We found a large variation in percent cytotoxicity [mean and (IQR) for ME/CFS and HC respectively, 34.1% (IQR 22.4–44.3%) and 33.6% (IQR 22.9–43.7%)] and no statistically significant differences between patients with ME/CFS and HC (p-value = 0.79). Analysis stratified on illness domain measured with standardized questionnaires did not identify an association of NK cytotoxicity with domain scores. Among all participants, NK cytotoxicity was not associated with survey results of physical and mental well-being, or health factors such as history of infection, obesity, smoking, and co-morbid conditions.

**Conclusion:**

These results indicate this assay is not ready for clinical implementation and studies are needed to further explore immune parameters that may be involved in the pathophysiology of ME/CFS.

**Supplementary Information:**

The online version contains supplementary material available at 10.1186/s12967-023-03958-2.

## Background

Myalgic encephalomyelitis/chronic fatigue syndrome (ME/CFS) is a complex multi-system illness of unknown etiology [1–3]. Diagnosis is based on presence of functional limitations associated with fatigue that is not significantly improved with rest, post-exertional malaise, unrefreshing sleep, and either orthostatic intolerance or cognitive problems. Additional symptoms may include pain in muscles and joints, headaches, and gastrointestinal issues. Symptoms can wax and wane in severity. Estimates place the prevalence of ME/CFS in the United States as afflicting around 1 million Americans (836,000 to 2.5 million) [4]. Unfortunately, while biologic abnormalities are recognized among patients with ME/CFS, none are specific enough for diagnostic use. This may be due to heterogeneity in the population afflicted with ME/CFS, including different yet unknown etiologies of the illness as well as fluctuations in symptom severity over time. Searching for disease etiology and biomarkers, researchers have investigated various pathogens, hormonal imbalances, genetic changes, metabolic disorders, and immune system dysfunction [5, 6].

Natural killer (NK) cell function is an area of particular interest. NK cells are lymphocytes of the innate immune system that, upon activation, rapidly respond to produce cytokines and to kill infected or neoplastic cells (cytotoxicity). They target cells lacking inhibitory ligands [e.g., major histocompatibility complex class I (MHC class-I)] and/or presenting activating ligands [e.g., MHC class-I chain-related protein A (MICA)]. NK cytotoxicity is mediated in part by release of cytoplasmic granules directed at the target cell. Perforin released from these granules creates channels in target cell membranes through which serine protease enzymes (granzymes) enter and activate caspases to induce apoptosis. NK function is measured in vitro by stimulation with activating ligands or co-culture with K562 cells, a myelogenous leukemia cell line that lacks MHC I. The readouts of these assays are cytokine production, cell membrane expression of activation markers, and killing of K562 target cells.

ME/CFS publications are inconsistent on whether NK function can be used to distinguish people with ME/CFS from those without; some finding no diminished cytotoxicity [7–10] and others finding this difference [11–16]. Mechanistically, some researchers find decreased perforin expression in people with ME/CFS while other find no difference relative to healthy controls [15, 17, 18]. These discrepancies may be due to small group sizes in some studies, differences in study populations, assay methods, and application of criteria to identify a person with ME/CFS. Nonetheless, the 2015 Institute of Medicine report on ME/CFS considered poor NK cell function to be a biomarker that correlates with severity of disease (IOM) [4].

Relatively few laboratories have contributed data to this question. Previously, investigators were limited to enrolling participants who could arrive at a clinic near a laboratory performing a NK cytotoxicity assay as same-day testing was required. In a prior publication, we showed that next-day testing of shipped blood specimens resulted in NK cytotoxicity measurements comparable to the gold-standard assay [19]. This allowed us to use centralized testing at a single laboratory to evaluate NK cytotoxicity in patients with ME/CFS compared to healthy controls enrolled from multiple clinics throughout the United States.

## Methods

### Recruitment

This study was conducted as part of the Multi-Site Clinical Assessment of Myalgic Encephalomyelitis/Chronic Fatigue Syndrome (MCAM) study [20]. The MCAM study enrolled participants from five specialty clinical sites located in the United States based on expert clinician diagnoses and used standardized assessment tools to characterize ME/CFS illness domains as previously described (sites: Mount Sinai Beth Israel, New York, New York; Institute for Neuro ImmuneMedicine, Miami and Fort Lauderdale, Florida; Bateman Horne Center, Salt Lake City, Utah; OpenMedicine Clinic, Mountain View, California; and Sierra Internal Medicine, Incline Village, Nevada) [20]. The MCAM cohort consists of patients with ME/CFS, healthy control participants (HC), and an ill comparison group (IC; patients with other fatigue-associated illnesses e.g., fibromyalgia, chronic Lyme disease). Participants were between the ages of 18 and 70 years at enrolment, roughly matching age (± 5 years) and sex between ME/CFS and HC groups. Matching IC was not done due to low enrollment of this group. All participants provided their consent for the main study conducted between 2012 and 2019. For the NK sub-study, participants signed an additional informed consent. NK testing was done between April 2017 and January 2019. MCAM and NK study protocols were approved by the Institutional Review Boards (IRB) of the Centers for Disease Control and Prevention (CDC), Western IRB for Open Medicine Institute Consortium, Mount Sinai Beth Israel, and Nova Southeastern University.

### NK testing

During a participant’s clinic visit, peripheral blood was drawn via antecubital venipuncture into heparinized tubes labeled with participant’s study identification number. Samples were shipped for next morning delivery in ambient temperature insulated packing. NK cell counts and NK cytotoxicity assays were performed on the day of shipment delivery at a flow cytometry lab (Flow Contract Site Laboratory, Bothell, WA, USA) under quality management systems using methods as previously described. [19] Briefly, NK cells (CD3− CD16/CD56 +) were measured by flow cytometry. NK cell cytotoxicity was performed with isolated peripheral blood mononuclear cells (PBMCs) incubated with K562 target cells at ratios of 100:1, 50:1, 25:1 and 0:1 (negative control) in triplicate and assessed with flow cytometry. The percent cytotoxicity for each sample replicate at each effector to target ratio (E:T) (100:1, 50:1 and 25:1) was calculated as percent of dead cells minus the average percent dead cells in the negative control replicates (E:T 0:1). Linear regression interpolated percent cytotoxicity at 50:1 with 95% confidence intervals.

### Clinical assessment data

We used data collected as part of the MCAM study to determine clinical characteristic of subjects [20]. The MCAM data collection did not always occur on the same day that blood was sampled for NK cell testing. For this analysis, we selected socio-demographics, illness onset (CDC Symptom Inventory, CDC-SI), symptom profile (CDC-SI), functional status [SF-36v2—Physical Component Summary (PCS)], sleep (Patient-Reported Outcomes Measurement Information System (PROMIS) Sleep Disturbance and Sleep-Related Impairment), pain (PROMIS Pain Interference and Pain Behavior), anxiety [Generalized Anxiety Disorder (GAD-7)], depression [Patient Health Questionnaire (PHQ-8)], and energy/fatigue (measured by MFI-20) [21–27] and measured time between data collection and NK cell sampling. Information on co-morbid conditions was obtained from the medical history form abstracted from medical records and reviewed by participants. For this analysis we included metabolic, autoimmune, and sleep conditions that might have associations with NK cell number or function: diabetes, hypoglycemia, thyroid abnormalities, fibromyalgia, gout, systemic lupus erythematosus, rheumatoid arthritis, Sjögren’s syndrome, other autoimmune diseases, sleep apnea, narcolepsy, and other sleep disorders (see Additional file 1: Table S2). Infection history was based on patient’s self-reported frequency of bacterial, viral, fungal, and other types of infections. Socio-demographics, CDC-SI, SF-36, PROMIS, MFI-20, co-morbid conditions, and physical exam were analyzed from the MCAM visit closest to NK sub-study enrollment.

### Statistical analysis

Statistical analysis was conducted primarily using SAS software (v.9.4, SAS Institute Inc., Cary, NC), but for better visualization some graphs were produced in R with packages epiR [v2.0.26, [28]] and ggplot2 [3.3.5, [29]]. Categorical variables were presented as frequency and percentage within group. Normality test and QQ-plot were performed to check the normality for continuous variables (NK count and cytotoxicity) and summary information of mean and standard deviation were also provided. The log-transformation was performed to improve normality of the NK variables. T-test was conducted to compare the mean of the NK variables between participants stratified by study groups and medical factors. The generalized linear model (GLM) was utilized for examining the bivariate association between NK measures and symptoms measures. As severity of illness could impact NK function, we stratified the participants with ME/CFS by median scores of ME/CFS group in Physical Component Summary (PCS) T-score of SF-36 as indicator of severity. Participants with PCS T-score ≤ 25.84 (the median of PCS T-scores in the ME/CFS group (n = 174)), were classified as “severe”, those above– as “less severe” ME/CFS (for comparison, 50 is about the mean of the US general population). Bonferroni correction was used to adjust p-values for the multiple group comparisons (“severe” ME/CFS, “less severe” ME/CFS, and HC groups). While the level of statistical significance was indicated by a p value < 0.05, the p-values for each comparison were also reported in tables.

## Results

**Fig. 1:**
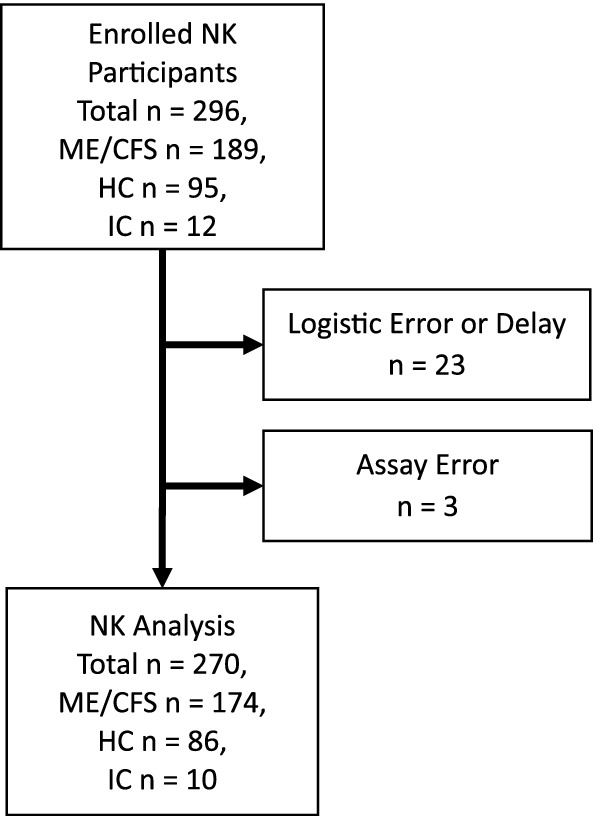
Participant Enrolment by Healthy Control (HC), Ill Control (IC), and Myalgic Encephalomyelitis/Chronic Fatigue Syndrome (ME/CFS) and Exclusions from Analysis

A total of 296 MCAM participants enrolled in the NK Sub-study (Fig. 1). Of these, 26 participants (n = 15 with ME/CFS, n = 9 HC, n = 1 IC) were excluded from NK analysis due to problems with the specimen (e.g., blood clots rendering specimens inadequate and delivery delayed beyond 24 h post-venipuncture). The remaining 270 specimens used for NK analysis came from 174 (64.5%) ME/CFS, 86 (31.8%) HC and 10 (3.7%) IC participants. Participants with ME/CFS enrolled in this sub-study varied by illness characteristics (Table 1) but were similar to the overall MCAM ME/CFS cohort [20].

**Table 1:** Characteristic of participants with ME/CFS (n = 174)—overall functioning and symptom status at intake

	Mean	Std. Dev.	Std. error	Min.	Lower quartile	Median	Upper quartile	Max.
**SF-36 (0–100)**								
Physical function	41.21	23.42	1.78	0.00	25.00	40.00	55.00	100.00
Role physical	16.13	28.95	2.21	0.00	0.00	0.00	25.00	100.00
Bodily pain	42.19	23.56	1.79	0.00	22.00	41.00	51.00	100.00
Vitality	19.77	16.92	1.29	0.00	5.00	15.00	30.00	80.00
General health	27.20	17.14	1.30	0.00	15.00	25.00	35.00	77.00
Role emotional	85.96	31.69	2.42	0.00	100.00	100.00	100.00	100.00
Social function	27.17	23.32	1.77	0.00	0.00	25.00	37.50	100.00
Mental health	68.02	18.93	1.44	16.00	56.00	72.00	84.00	96.00
**MFI-20 (0–20)**								
General fatigue	17.95	2.46	0.19	9.00	17.00	19.00	20.00	20.00
Physical fatigue	17.31	2.69	0.20	6.00	16.00	18.00	20.00	20.00
Reduced activity	15.77	3.56	0.27	5.00	14.00	16.00	19.00	20.00
Reduced motivation	11.62	3.83	0.29	4.00	9.00	12.00	14.00	20.00
Mental fatigue	14.37	3.70	0.28	4.00	12.00	15.00	17.00	20.00
**CDC-SI**								
Fatigue duration (yrs)	13.62	10.42	0.80	0.25	5.00	12.00	21.00	61.00
Number of CFS symptoms	5.76	2.02	0.16	0.00	5.00	6.00	7.00	8.00
CFS symptom score	50.99	24.01	1.87	0.00	36.25	51.50	66.50	115.50
**PROMIS (T-score)**								
Sleep disturbance	58.45	7.86	0.60	38.00	54.30	58.30	63.70	76.50
Sleep Related impairment	60.73	8.22	0.62	30.00	56.10	61.30	66.30	80.00
Pain intensity	59.56	9.09	0.69	41.00	56.10	60.90	65.50	78.30
Pain behavior	56.01	7.88	0.61	36.70	54.80	58.60	60.60	68.30

The demographic characteristics of participants in the NK cell function study are shown in Table 2. The ME/CFS and HC groups were similar in age, sex and education, but differed in race/ethnicity, employment, insurance and marital status. IC results were excluded from statistical comparison to the other groups due to insufficient enrolment numbers. The ME/CFS and HC groups differed significantly by race (p-value < 0.0001), with more White participants in the ME/CFS group. Participants with ME/CFS were less likely to report being married or in a committed relationship. Participants with ME/CFS were less likely to be employed full-time, but also were more likely not to report employment status. Finally, participants with ME/CFS were slightly more likely to have insurance.

**Table 2:** Description of the study sample

	ME/CFS (n = 174)		HC (n = 86)		IC (n = 10)	
	n	%	n	%	n	%
**Age (years)**						
18–29	17	9.77	15	17.44	0	0
30–39	26	14.94	13	15.12	1	10.00
40–49	28	16.09	10	11.63	1	10.00
50–59	46	26.44	31	36.05	5	50.00
≥ 60	57	32.76	17	19.77	3	30.00
**Sex**						
Male	50	28.74	28	32.56	2	20.00
Female	124	71.26	58	67.44	8	80.00
Race**						
White	153	87.93	53	61.63	10	100.00
Black	1	0.57	4	4.65	0	0
All others	11	6.32	20	23.26	0	0
Missing	9	5.17	9	10.47	0	0
Ethnicity**						
Hispanic	10	5.75	25	29.07	0	0
Non-Hispanic	146	83.91	51	59.30	8	80.00
Missing	18	10.34	10	11.63	0	0
Marital status*						
Married/committed	84	48.28	56	65.12	8	80.00
Previously married	30	17.24	13	15.12	1	10.00
Never married	57	32.76	17	19.77	1	10.00
Missing	3	1.72	0	0	0	0
Employment**						
Full-time	22	12.64	49	56.98	4	40.00
Part-time	19	10.92	17	19.77	1	10.00
Not working	109	62.64	17	19.77	3	30.00
Missing	24	13.79	3	3.49	2	20.00
Insurance*						
Yes	169	97.13	77	89.53	10	100.00
No	5	2.87	9	10.47	0	0
**Education**						
Less than high school	1	0.57	2	2.33	0	0
High school graduate	43	24.71	22	25.58	5	50.00
College graduate	73	41.95	27	31.40	1	10.00
Post college	56	32.18	34	39.53	4	40.00
Missing	1	0.57	1	1.16	0	0
**Illness onset**						
Gradual	47	29.94	NA	NA	NA	NA
Sudden	110	70.06	NA	NA	NA	NA

For ME/CFS and HC comparison: *p-value < 0.05, **p-value < 0.0001

**Fig. 2:**
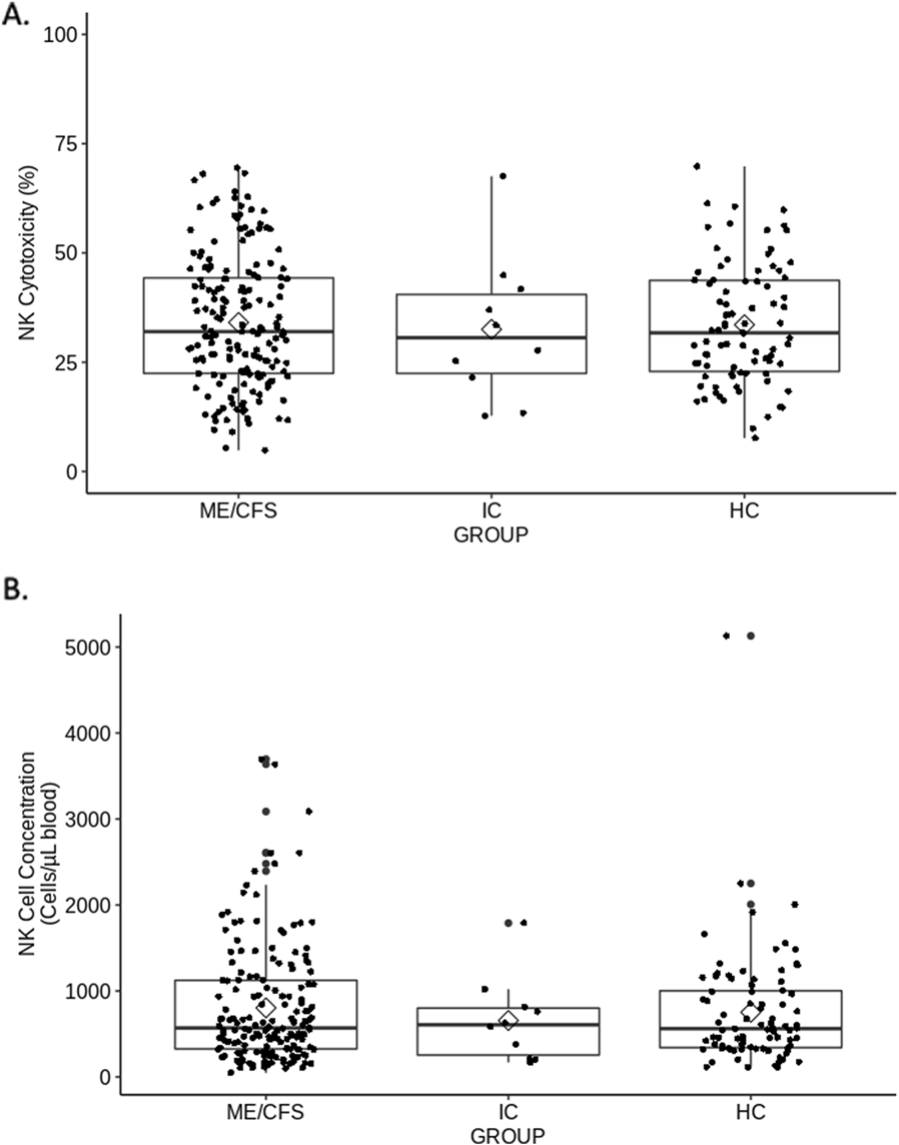
Box and whisker plots of Natural Killer (NK) Cell Cytotoxicity and Concentration by Illness Group **A** NK cytotoxicity **B** NK cell concentration in blood. The top and bottom of the boxes are the 25% and 75% quartiles of NK cytotoxicity. The horizontal lines within the box are the medians, and the diamonds are the means. The points are individual participant results

Figure 2A and B display the distribution of the NK data across three study groups [Mean cytotoxicity and 25–75% Inter-quartile range (IQR): ME/CFS 34.1% (IQR 22.4–44.3%); IC 32.5% (IQR 22.4–40.5%); HC 33.6% (IQR 22.9–43.7%)] or in NK cell concentration in blood: ME/CFS 802.7 cell/µL (IQR 325.8–1123.3); IC 656.0 cell/µL (IQR 254.1–800.0); HC 751.4 cell/µL (IQR 340.8–1002.1). No statistically significance was found in NK cytotoxicity (p-value = 0.79) and NK cell count (p-value = 0.91) between ME/CFS and HC groups.

The mean NK cytotoxicity did not significantly differ among “severe ME/CFS”, “less severe ME/CFS” and healthy control groups [Severe ME/CFS group mean (SE) = 34.70 (1.58)]; less severe ME/CFS group 33.48 (1.57); HC group 33.57 (1.58); Bonferroni adjusted p-value = 1]. Similarly, no statistically significant difference was found in the mean NK cytotoxicity between “Sudden onset ME/CFS”, “Gradual onset ME/CFS” and healthy control groups [Sudden onset ME/CFS group mean (SE) = 34.86 (1.51); Gradual onset ME/CFS 33.18 (2.22); Bonferroni adjusted p-value = 0.75] or between “ME/CFS with long illness duration” (≥ 10 yrs) (n = 126), “ME/CFS with moderate illness duration” (4–10 yrs) (n = 30), “ME/CFS with short illness duration” (< 4 yrs) (n = 13)” and healthy control groups [ME/CFS with long illness duration group mean (SE) = 33.81 (1.36); ME/CFS with moderate illness duration ME/CFS 35.04 (2.80); with short illness duration 37.44 (4.51); Bonferroni adjusted p-value = 0.69].

We examined correlations of NK cytotoxicity measures with measures of symptoms [subscale scores of SF-36, MFI-20, CDC-SI and PROMIS—Additional file 1: Table S1] and did not identify any significant associations. The mean time interval between questionnaire data used to evaluate correlation with NK cytotoxicity and date of sample collection for NK cell assays was 26 days (median = 7). Time interval between data collection and collection of blood sample for NK cell testing was evaluated as a covariate for correlations between measures of NK cytotoxicity and symptoms and was not significant (data not shown).

NK cell counts and cytotoxicity stratified by medical factors and co-morbid medical conditions (Additional file 1: Table S2) did not identify significant differences with one exception. Participants with a history of frequent viral infections had a lower NK count than those without a history of viral infections (mean cell/µL = 534.96, SE = 55.47 versus mean cell/µL = 826.82, SE = 48.76; p-value = 0.0080).

## Discussion

This is the first US study of NK cell function and counts in participants with ME/CFS and healthy controls enrolled from multiple clinics and assayed without a freezing step in an independent central laboratory. The study found a large distribution in percent cytotoxicity for both groups [mean and (IQR) for ME/CFS and HC respectively, 34.1% (IQR 22.4–44.3%) and 33.6% (IQR 22.9–43.7%); p-value = 0.79]. Similarly, mean NK cell counts did not differ [p-value = 0.91]. Further stratified analyses based on symptom measured with standardized questionnaires did not identify an association of NK cytotoxicity with domain scores.

As noted, findings from prior studies varied. Several studies failed to find an association of ME/CFS and reduced NK cytotoxicity. A previous large study based on samples from the UK biobank found no difference in functional markers [intracellular IFN-γ or cell surface expression of CD25 or CD107a] using cryopreserved PBMCs from ME/CFS patients and healthy controls stimulated with K562 cells or cross-linking antibody to CD16 [7]. While we found cryopreservation to be detrimental to NK cell functional assays [19], we did not incorporate the same incubation steps used by Cliff et al.[7]. Other studies have not found a difference between ME/CFS patients and healthy individuals in measures of PBMC NK cell killing of K562 cells [8–10]. In contrast, three ME/CFS research groups reported decreased NK cytotoxicity in ME/CFS patients when measured in PBMCs or whole blood. [11–16, 30, 31]. Another study found variable NK cell cytotoxicity results comparing patients with ME/CFS to control [32].

This study has some limitations that curtail the strength of the findings. Comparison between participant characteristics and NK measures were limited due to the timing and method of collection of some data. Ideally, questionnaire data used to evaluate symptoms and the blood sample for NK cell evaluation would be collected on the same day. In our study the mean time interval was 26 days (median = 7). Changes in these characteristics during the interval between data collection and blood draw for testing could mask an association that exists between contemporaneous measures. However, we evaluated the time effect on the association by including the time interval of the data collection as a covariate, we did not find any significant time effect. Self-reported measures of infections have not been validated but were asked as an initial exploration to link NK function to illness history. Overall, in the combined sample of ME/CFS and HC, history of viral infections (Additional file 1: Table S2) was associated with decreased NK cell blood concentration. However, the difference is unlikely to be clinically significant as the NK cell counts were within the normal range in both strata. Furthermore, this self-reported measure is subject to recall bias, the definition of “frequent” was subjective, extent of medical record documentation varied and both NK counts were in normal range, limiting clinical value. The number of participants in the IC group was too small for a meaningful comparison. Finally, in the validation study, PBMC samples that were tested both on the same and next day did demonstrate a loss in NK cell function [19]. We cannot entirely exclude that next day testing of isolated PBMCs impairs NK cell cytotoxicity to the extent that group differences would be obscured. However, the full range of activity observed in patients and controls make this explanation less likely.

The strengths of this study include the large numbers of well characterized patients enrolled from multiple clinics and the ability to test fresh samples in a central laboratory with an assay validated against a gold standard assay. Our findings do not enitrely exclude an association between ME/CFS and decreased NK cell function as NK functional measures could be important in a subset of patients or be a useful marker of response to therapy. However, failure to see statistically significant group differences in analyses stratified by disease severity makes this explanation less likely. Establishing a clinically useful NK cytotoxicity assay would require additional study to establish robust thresholds for normal values as well as to evaluate the impact of age and race on these values. In addition, studies confirming test–retest validity and the impact of intercurrent variables such as diurnal fluctuations, lack of sleep or stress need to be conducted. Further, it should be noted that even in a research setting, nearly 9% of samples were inadequate for analysis. This study suggests NK cell cytotoxicity reflected in this validated assay is not a robust and reliable indicator of ME/CFS. While loss of NK cytotoxicity associated with next-day testing cannot be completely excluded as an explanation for failure to find an association of decreased NK cell function with ME/CFS, requiring same day testing would effectively preclude establishing this as a useful clinical test in most settings that do not have access to such a lab. Further studies are needed to explore immune parameters that may be involved in the pathophysiology of ME/CFS.

## Conclusion

In conclusion, these results indicate this natural killer cell assay is not ready for clinical implementation and studies are needed to further explore immune parameters that may be involved in the pathophysiology of ME/CFS.

## Supplementary Information

**Additional file 1: Table S1.** Correlation of NK Cytotoxicity by Measures of Symptoms among Study Participants (n = 174 with ME/CFS, n = 86 healthy controls; excluding ill comparison group, n = 10) [total n = 260]. **Table S2.** NK Count and NK Cytotoxicity by Medical Factors and Co-Morbid Conditions among Study Participants (n = 174 with ME/CFS, n = 86 healthy controls; excluding ill comparison group, n = 10).

## Data Availability

The raw data supporting the conclusions of this article will be made available mailto:without undue reservation. Requests should be sent to the MECFS program mailbox (https://www.cdc.gov).

## Abbreviations

CDC-SI: CDC symptom inventory
E:T: Effector to target ratio
GAD-7: Generalized anxiety disorder
HC: Health control
IC: Ill comparison
IQR: Inter-quartile range
MCAM: Multi-site clinical assessment of ME/CFS
ME/CFS: Myalgic encephalomyelitis/chronic fatigue syndrome
MFI-20: Multidimensional fatigue inventory 20
NK: Natural killer
PHQ-8: Patient Health Questionnaire 8
PCS: Physical Component Summary
PROMIS: Patient-Reported Outcomes Measurement Information System
SE: Standard error
SF-36: Short form 36
