# Supplementary material for: Natural killer cytotoxicity in myalgic encephalomyelitis/chronic fatigue syndrome (ME/CFS): a multi-site clinical assessment of ME/CFS (MCAM) sub-study
Source: J Transl Med. 2023 Apr 3;21:242. doi: 10.1186/s12967-023-03958-2 (PMC10069115; doi:10.1186/s12967-023-03958-2)
Supplement: Supplementary file 1 — Additional file 1: Table S1. Correlation of NK Cytotoxicity by Measures of Symptoms among Study Participants (n = 174 with ME/CFS, n = 86 healthy controls; excluding ill comparison group, n = 10) [total n = 260]. Table S2. NK Count and NK Cytotoxicity by Medical Factors and Co-Morbid Conditions among Study Participants (n = 174 with ME/CFS, n = 86 healthy controls; excluding ill comparison group, n = 10). [file 12967_2023_3958_MOESM1_ESM.docx]

**Table S1. Correlation of NK Cytotoxicity by Measures of Symptoms among Study Participants** **(n= 174 with ME/CFS, n=86 healthy controls; excluding ill comparison group, n=10) [total n = 260].**

|  |  | NK Count (cells/µL) | | | NK Cytotoxicity (% target cell death) | | |
| --- | --- | --- | --- | --- | --- | --- | --- |
|  | Parameter | Coefficient Estimate | Standard Error | p-value | Coefficient Estimate | Standard Error | p-value |
| SF-36v2 | Physical Component Score (PCS) | -0.0543 | 2.6089 | 0.9834 | 0.0029 | 0.0568 | 0.9600 |
|  | Mental Component Score (MCS) | -3.1654 | 3.5658 | 0.3755 | 0.0084 | 0.0777 | 0.9137 |
|  | Physical Function | -0.6252 | 3.0214 | 0.8362 | 0.0394 | 0.0658 | 0.5491 |
|  | Role Physical | -1.4338 | 2.7028 | 0.5962 | -0.0203 | 0.0589 | 0.7308 |
|  | Bodily Pain | 2.0095 | 3.2708 | 0.5395 | 0.0185 | 0.0715 | 0.7964 |
|  | Vitality | -0.3816 | 2.8144 | 0.8923 | -0.0055 | 0.0615 | 0.9290 |
|  | General Health | -1.7833 | 3.0094 | 0.5540 | -0.0003 | 0.0656 | 0.9965 |
|  | Role Emotional | -4.1460 | 3.1556 | 0.1901 | 0.0225 | 0.0689 | 0.7448 |
|  | Social Function | -2.0411 | 2.5905 | 0.4315 | -0.0024 | 0.0565 | 0.9662 |
|  | Mental Health | -0.1849 | 3.8267 | 0.9615 | 0.0388 | 0.0835 | 0.6428 |
| MFI-20 | General Fatigue | 0.3885 | 7.5521 | 0.9590 | 0.0014 | 0.1691 | 0.9932 |
|  | Physical Fatigue | 4.1047 | 6.3955 | 0.5216 | -0.0248 | 0.1562 | 0.8740 |
|  | Reduced Activity | -3.0913 | 7.5288 | 0.6817 | 0.0404 | 0.1688 | 0.8112 |
|  | Reduced Motivation | -7.4725 | 9.2080 | 0.4178 | 0.0288 | 0.2068 | 0.8895 |
|  | Mental Health | 0.1748 | 8.4969 | 0.9836 | 0.1377 | 0.1903 | 0.4699 |
| CDC-SI | Number of CFS Symptoms | 2.6322 | 15.7253 | 0.8672 | -0.0572 | 0.3415 | 0.8671 |
|  | CFS Symptom Score | 0.6141 | 1.6004 | 0.7016 | -0.0011 | 0.0348 | 0.9752 |
| PROMIS | Sleep Disturbance | 0.2971 | 4.2891 | 0.9448 | 0.0243 | 0.0936 | 0.7951 |
|  | Sleep Related Impairment | 0.6812 | 3.5842 | 0.8494 | 0.0047 | 0.0782 | 0.9517 |
|  | Pain Interference | 0.9868 | 4.0734 | 0.8088 | -0.1010 | 0.0886 | 0.2554 |
|  | Pain Behavior | 2.5850 | 4.1819 | 0.5370 | -0.0643 | 0.0914 | 0.4825 |
| PHQ-8 | Depression Score | -0.5177 | 8.7299 | 0.9528 | 0.1456 | 0.1909 | 0.4466 |
| GAD-7 | Anxiety Score | -0.1156 | 11.2779 | 0.9918 | 0.1400 | 0.2387 | 0.5583 |

**Table S2.** **NK Count and NK Cytotoxicity by Medical Factors and Co-Morbid Conditions among Study Participants (n= 174 with ME/CFS, n=86 healthy controls; excluding ill comparison group, n=10).**

|  |  |  |  | NK Count (cells/µL) | | | NK Cytotoxicity (% target cell death) | | |
| --- | --- | --- | --- | --- | --- | --- | --- | --- | --- |
|  |  |  | n | Mean | Standard Error | p-value | Mean | Standard Error | p-value |
| Infection History | Viral | Yes | 42 | 534.96 | 55.47 |  | 32.89 | 1.76 |  |
|  |  | No | 198 | 826.82 | 48.76 | 0.01 | 34.19 | 1.08 | 0.60 |
| Obesity | BMI≥30 | Yes | 53 | 756.65 | 108.37 | 0.36 | 34.26 | 1.86 | 0.89 |
|  |  | No | 184 | 816.49 | 48.71 |  | 33.94 | 1.10 |  |
| Smoking Status | Current smoker | Yes | 6 | 576.81 | 129.77 | 0.72 | 31.13 | 6.38 | 0.65 |
|  |  | No | 242 | 769.74 | 39.98 |  | 33.79 | 0.92 |  |
| Medical conditions | Diabetes (type 1 or 2) | Yes | 10 | 1232.77 | 451.96 | 0.14 | 38.57 | 5.12 | 0.31 |
|  |  | No | 249 | 769.74 | 39.41 |  | 33.75 | 0.93 |  |
|  | Hypoglycemia | Yes | 33 | 730.52 | 84.25 | 0.94 | 35.20 | 2.73 | 0.60 |
|  |  | No | 226 | 795.95 | 46.24 |  | 33.76 | 0.97 |  |
|  | Thyroid conditions^&^ | Yes | 56 | 897.32 | 93.05 | 0.17 | 33.03 | 2.03 | 0.60 |
|  |  | No | 203 | 757.35 | 46.54 |  | 34.19 | 1.02 |  |
|  | Thyroid condition is under control | Yes | 27 | 703.78 | 118.01 | 0.37 | 29.95 | 2.80 | 0.14 |
|  |  | No | 232 | 797.37 | 44.55 |  | 34.40 | 0.96 |  |
|  | Fibrositis or Fibromyalgia | Yes | 79 | 797.23 | 72.20 | 0.92 | 33.40 | 1.74 | 0.69 |
|  |  | No | 180 | 783.40 | 51.13 |  | 34.18 | 1.07 |  |
|  | Gout | Yes | 2 | 531.62 | 33.67 | 0.88 | 27.37 | 0.70 | 0.53 |
|  |  | No | 257 | 789.61 | 42.03 |  | 33.99 | 0.92 |  |
|  | Systemic Lupus Erythematosus | Yes | 2 | 679.60 | 360.65 | 0.88 | 32.04 | 14.93 | 0.85 |
|  |  | No | 257 | 788.46 | 42.00 |  | 33.95 | 0.91 |  |
|  | Rheumatoid arthritis | Yes | 3 | 838.66 | 201.06 | 0.52 | 40.20 | 8.20 | 0.46 |
|  |  | No | 256 | 787.02 | 42.17 |  | 33.87 | 0.92 |  |
|  | Sjogren’s syndrome | Yes | 7 | 582.55 | 128.59 | 0.62 | 38.39 | 7.93 | 0.42 |
|  |  | No | 252 | 793.31 | 42.71 |  | 33.82 | 0.91 |  |
|  | Autoimmune disease | Yes | 36 | 786.93 | 106.11 | 0.94 | 35.22 | 2.75 | 0.57 |
|  |  | No | 223 | 787.73 | 45.43 |  | 33.73 | 0.96 |  |
|  | Sleep Apnea | Yes | 38 | 634.88 | 76.97 | 0.15 | 33.75 | 2.09 | 0.93 |
|  |  | No | 221 | 813.88 | 46.91 |  | 33.97 | 1.01 |  |
|  | Narcolepsy | Yes | 3 | 489.53 | 86.41 | 0.66 | 20.40 | 3.69 | 0.11 |
|  |  | No | 256 | 791.11 | 42.16 |  | 34.10 | 0.92 |  |
|  | Other sleep disorder | Yes | 40 | 751.60 | 85.78 | 0.87 | 33.00 | 2.37 | 0.66 |
|  |  | No | 219 | 794.19 | 46.86 |  | 34.11 | 0.99 |  |

^&^ Thyroid conditions include hyperthyroidism and hypothyroidism
